# Effects of Intranasal Oxytocin on Emotion Regulation in Insecure Adolescents: Study Protocol for a Double-Blind, Randomized Controlled Trial

**DOI:** 10.2196/resprot.5643

**Published:** 2016-11-02

**Authors:** Monika Szymanska, Carmela Chateau Smith, Julie Monnin, Patrice Andrieu, Frédérique Girard, Lucie Galdon, Marie Schneider, Lionel Pazart, Sylvie Nezelof, Lauriane Vulliez-Coady

**Affiliations:** ^1^Laboratory of Clinical and Integrative Neuroscience EA481University of Franche-ComteBesançonFrance; ^2^UFR SVTECOMUE Bourgogne Franche-ComtéUniversity of BurgundyDijonFrance; ^3^Clinical Investigation Center, CIC-IT 808, INSERMUniversity Regional HospitalUniversity of Franche-ComteBesançonFrance; ^4^Department of Child and Adolescent PsychiatryUniversity Regional HospitalBesançonFrance; ^5^Clinical Investigation Center, CIC-IT 1431, INSERMUniversity Regional HospitalBesançonFrance

**Keywords:** intranasal oxytocin, attachment, adolescents, parent-adolescent interaction, randomized controlled trial

## Abstract

**Background:**

Emotional dysregulation and impaired attachment are potential contributors to the development of psychopathology in adolescence. This raises the question of whether oxytocin (OT), the paradigmatic “attachment hormone,” may be beneficial in such contexts. Recent evidence suggests that intranasal administration of OT increases affiliative behavior, including trust and empathy. OT may also facilitate social reciprocity by attenuating the stress response to interpersonal conflict. To date, few studies have investigated the effects of intranasal oxytocin (IN-OT) on neurophysiological emotion regulation strategies in healthy adolescents, particularly during parent-adolescent interaction. To understand these mechanisms, our study will examine the effects of IN-OT on emotion regulation in adolescents during parent-adolescent stressful interactions, and on each adolescent’s visual and neurophysiological strategies when visualizing attachment-related pictures. We hypothesize that IN-OT will influence psychophysiological outcomes under conditions of stress. We predict that IN-OT will momentarily increase feelings of safety and attenuate stress and hostile behavior during conflict situations. OT may also enhance attachment security by increasing comfort and proximity-seeking, and reducing neurophysiological hyperactivation.

**Objective:**

The objective of this study is to evaluate the effects of IN-OT on insecure adolescents by studying their behavior and discourse during a disagreement with one of their parents. Their neurophysiological responses to pictures eliciting attachment-related emotions and their visual exploration strategies will also be investigated.

**Methods:**

In this randomized, double-blind, placebo-controlled parallel-group design, 60 healthy male adolescents classified as insecurely attached will receive 24 international units (IU) of IN-OT versus placebo (PB), 45 minutes before the experimental tasks. Each adolescent will then be invited to engage in an experimental conflict discussion with one of his parents. The conflict session will be videotaped and coded for verbal and non-verbal interaction behavior, using the Goal-Corrected Partnership in Adolescence Coding System (GPACS). Each adolescent will then be asked to visualize attachment-related pictures on a screen. Eye-tracking (ET) and neurophysiological responses, including electrodermal activity (EDA) and heart rate (HR), will be recorded simultaneously and continuously during attachment-related picture viewing (Besançon Affective Picture Set-Adolescents, BAPS-Ado).

**Results:**

Enrollment for the study was completed in May 2016. Data analysis commenced in July 2016. Study results will be submitted for publication in the winter of 2017.

**Conclusions:**

OT is a complex molecule with many facets that are not yet fully understood. This experimental protocol will increase scientific and clinical knowledge of emotion regulation skills in insecure adolescents by assessing the impact of IN-OT on parent-adolescent interaction and on the visual processing of attachment-related emotions. Positive results could lead to therapeutic uses of IN-OT to treat emotion dysregulation in adolescence.

## Introduction

### Background

Adolescence is a period of psychobiological and social changes necessary to achieve psychological maturation and develop autonomy [1,2]. However, because of their history and temperament, some adolescents will have difficulty regulating their emotional reactivity, which could lead to emotional dysregulation and mental suffering when emotions are too intense or violent. This emotional dysregulation can be associated with psychopathology, such as depression [3], eating disorders [4], substance abuse [5], or antisocial and delinquent behavior [6]. In recent years, the impact of attachment bonds on adolescent emotional (dys)regulation has been seriously questioned.

Attachment has been defined as an innate psychobiological system that motivates people to seek proximity in times of distress [7-9]. The quality of this system is determined by early caregiving experiences and results in individual differences in attachment security. Harmonious attachment experiences can result in secure attachment, while disordered experiences can lead to insecure (ie, avoidant or anxious) or disorganized attachment [10]. Attachment patterns become part of the general interpersonal style that will influence strategies of closeness-distance regulation toward the attachment figure, as well as strategies of emotion regulation [11,12].

Despite the change in parent-child interaction during the process of adolescence, where more distance and more conflict linked with autonomy occur, parents remain a secure base for the adolescent in times of distress. It is expected that parents will continue to offer a protective base to help adolescents to regulate their emotions and to sustain a “goal-directed partnership” with them. This relationship context, in which adolescents might experience disagreement situations and develop a variety of emotional responses to deal with potentially elicited distress, is linked to the attachment style of the adolescent [13-16].

Social attachment interaction has health benefits, and its absence can be associated with both physical and mental illness, with broad consequences throughout the lifespan [17]. Researchers have shown that a securely attached adolescent reports less conflict with parents and fewer psychopathological symptoms [18-20]. During conflictual exchange, secure adolescents display collaborative communication, while also expressing anger and hostility. In this context, secure adolescents feel safe to communicate differences of opinions and vulnerabilities with trust and openness [14]. In contrast, insecurely attached, avoidant adolescents display deflecting and/or minimizing attachment strategies during interaction with their parents. They tend to manifest distracted and disengaged behavior and to elaborate on their disagreement, maintaining emotional distance, instead of reciprocity. Adolescents with ambivalent attachment may present entangled and/or oscillatory interactions. Here, the adolescent may manifest distressed or frustrated behavior when facing awkward reciprocity and parental inconstancy [21,22]. These two insecure attachment patterns could become risk factors for emotional dysregulation linked to a broad spectrum of psychopathology (eg, anxiety, depression, conduct disorders). Finally, disorganized parent-adolescent dyadic interaction is characterized by the inability to collaborate, marked by an unbalanced relationship, characterized by different profiles, such as role-confusion or hostile/passive patterns [23].

Recent findings suggest that a common neurobiological system appears to underlie attachment via release of various neuropeptides, the main one being oxytocin (OT) [24]. OT, synthesized in the magnocellular neurons of the supraoptic and paraventricular nuclei of the hypothalamus, projects to the posterior pituitary [25], where it is released into the bloodstream causing a wide range of bodily effects [26-28]. Within the brain, OT can act as a neurotransmitter and/or neuromodulator in various limbic, midbrain, and hindbrain structures [29]. Its central neuromodulatory role in sexual behavior [30], lactation, and childbirth [31] is increasingly recognized. Due to its role in parent-child bonding [32], empathy [24,33,34], trust [35,36], and the promotion of social behavior [37], OT has earned a strong popular and scientific reputation as the “hormone of love” or “hormone of attachment” [38].

These discoveries have led researchers to investigate the effects of intranasal oxytocin (IN-OT) within the healthy [39-45] and clinical [46-49] population. However, it appears that IN-OT can be more beneficial for some individuals than for others, and that the effects of IN-OT seem to be moderated by contextual factors (eg, presence of stranger versus friend [50]), and individual factors (eg, differences in attachment [39,43,44,51-57]). Individual attachment differences have proved to be a strong modulator of OT action [49,55,56], and mediate stress-buffering effects in times of stress [58]. Interestingly, the effects of OT appear to be moderated by attachment anxiety level [40]. In highly preoccupied anxious individuals, OT appears to exacerbate interpersonal insecurity and to affect how the quality of maternal interaction is remembered. After IN-OT administration, highly preoccupied individuals remembered their mothers as less caring and less close, while less anxiously attached individuals remembered their mothers as more caring and more close [40]. The level of attachment avoidance also seems to influence IN-OT effects. In highly avoidant individuals, IN-OT has been shown to increase constructive interaction [52]. For example, under the placebo (PB) condition, avoidant males trusted an unknown protagonist less, feared betrayal more, and decided less often to approach their protagonist cooperatively. However, these effects of attachment avoidance disappeared when males received IN-OT prior to decision-making. Participants high in attachment avoidance show more trust, lower betrayal aversion, and they cooperated more when given IN-OT rather than PB. Other findings [44] have shown that individual differences in attachment act as an endophenotype that moderates the effects of the OT system on social behaviors and cognition following social exclusion. OT appears to contribute to ongoing cooperation with a rejecting but initially cooperative partner, but only for those with low attachment avoidance [44]. Recent research [39] has shown that IN-OT increases communion skills in avoidantly attached individuals, who were especially likely to perceive themselves as more kind, warm, and gentle after receiving IN-OT than after receiving the PB. There was also a major effect of IN-OT on agency [59] in anxiously attached individuals, who showed a selective decrease in independent, self-confident behavior following IN-OT administration. This variability in IN-OT effects raises many questions and controversies regarding the effects of this hormone on social interaction, and especially on the influence of moderating factors. This lack of consensus together with many inconsistent results have led researchers [60-62] to openly question the true impact either of IN-OT in general or of its potential therapeutic effect [63]. Thus, to better understand the effects of OT and the impact of moderating factors on the mechanisms of socio-emotional interaction, we propose an original study protocol that provides new avenues of research into OT from both social and emotional perspectives.

### Current Study

This study protocol seeks to examine the effects of IN-OT administration on emotion regulation strategies in insecure adolescents. A reliable protocol is crucially needed in the field of attachment and OT research, if we want to build a solid theoretical background for interpreting OT, before IN-OT can be proposed as adjuvant therapy in insecure adolescents with emotion dysregulation. We hypothesize that IN-OT will promote a “momentary” subjective experience of attachment safety and proximity-seeking in insecurely attached adolescents during dyadic stressful interaction. We suppose that both anxious and avoidant adolescents under treatment will communicate with their parents with more self-disclosure, and will manifest less negative (eg, controlled, hostile, odd) behavior than under the PB condition. We suppose that IN-OT will positively bias parent-adolescent interaction, especially for highly anxious individuals, because it should attenuate their chronic concerns about distress, separation, and abandonment. OT might support parent-adolescent interaction and emotion regulation for highly avoidantly insecure adolescents (often suspicious and defensive), who avoid expressing emotions in the family context. We hypothesize that, in general, insecurely attached adolescents under IN-OT will process attachment-related emotional information in a more accessible and open way compared to PB conditions. Compared to the PB, IN-OT will increase exploration of distress and comfort pictures (eg, first fixation duration; number and duration of fixations). During visualization of distress pictures, IN-OT will attenuate emotional arousal by decreasing neurophysiological reactivity (amplitude of specific skin conductance response, SCR), and should also decrease SCR and decelerate heart rate (HR).

The main objective is to evaluate the effect of IN-OT on the behavior and discourse of insecure adolescents during a disagreement (stressful situation) with one of their parents. This evaluation will be based on the average score on the scales (4 negative and 1 positive) of the Goal-Corrected Partnership in Adolescence Coding System (GPACS) (personal communication from K Lyons-Ruth, author of the GPACS). Secondary objectives will be to evaluate the effect of IN-OT versus PB on visual exploration strategies for images eliciting attachment-related emotions (ie, distress and comfort). This characterization will be based on the study of eye parameters (ie, total picture fixation time, and average number of fixations per emotion) assessed by a remote eye tracking (ET) device.

We are conducting a randomized, double-blind, placebo-controlled protocol in which insecure adolescent male participants received IN-OT or PB. The protocol was designed to investigate the effects of IN-OT on emotion regulation strategies, by testing whether attachment anxiety and avoidance moderate the effects of IN-OT during a conflict discussion with a parent, and during the visualization of attachment-related pictures.

Emotion (dys)regulation strategies will be evaluated through a multi-dimensional approach: ET and neurophysiological measurements including electrodermal activity (EDA) and HR. These neurophysiological measurements have recently received a great deal of attention as potential biological markers of individual differences in affective response [64,65]. HR reflects the continuous interplay between the sympathetic and the parasympathetic nervous systems and is regarded as a measure of autonomic flexibility and even as a biological marker of emotional response [65]. SCR, a form of EDA, is supposed to primarily reflect autonomic arousal, regardless of the state induced by the stimulus, whether negative or positive [64]. Changes in SCR represent activity within the sympathetic axis of the autonomic nervous system (ANS), measured by autonomic innervations of the skin sweat glands [66]. The neurobiological underpinnings of SCR are widespread and not exclusively related to the defense network. Likewise, HR is also under the control of the ANS [67]. Deceleration of HR is often shown in response to affective stimuli, and is more pronounced in reaction to negative stimuli [67]. HR deceleration seems to be more closely related to attentional processing regarding changes in affective states rather than to valence or arousal [68]. In summary, SCR and HR represent different neural systems, thus allowing us to assess more accurately the effects of OT in response to attachment-related emotions.

## Methods

### Type of Clinical Trial

We present a randomized, double-blind, placebo-controlled, parallel clinical trial protocol for IN-OT versus PB. We will argue in favor of a multi-method, multi-dimensional (individual and interpersonal strategies) approach to emotion-regulation assessment. We will use two types of attachment paradigms: a conflict discussion with the parent (attachment figure), and the visualization of standardized attachment-related emotional pictures (ie, distress, comfort, joy-complicity, and neutral stimuli) to assess behavioral, neuropsychological, and neurophysiological responses. Although some studies [69-72] have underlined the importance of investigating the effects of IN-OT on adult females, given the risk factors (eg, uterine contraction and menstrual cycle disturbance) for female adolescents in non-clinical environments, this experimental research will be conducted exclusively on healthy male adolescents.

### Study Setting

This study will be conducted in Besançon, France at the University Regional Hospital, in the Department of Adolescent and Child Psychiatry.

### Ethical Criteria

The study will be carried out in accordance with the principles of the Helsinki Declaration. This protocol is governed by French legislation concerning interventional biomedical research and was submitted to the local ethics committee (ie, Comité de Protection des Personnes-EST II) and approved in 2013 (number 2013-000029-29). The study was also approved by the French Agency for the Safety of Health Products (ie, Agence Nationale de Sécurité du Médicament et des Produits de Santé, ANSM) in 2013. The trial is registered with the Clinical Trials Register (NCT02301715). An anonymous identification code will be attributed to each participant in the study, and the list identifying participating patients with their personal data will be stored by the investigator and kept strictly confidential.

### Participants

For this study, 60 French-speaking male adolescents, aged 13 to 20 years, each accompanied by one parent, will be recruited from secondary schools in Besançon, France. Compensation will be given to each adolescent (a €20 gift voucher, Ticket Kadéos Universel, Edenred, France) and to the parent accompanying him (a €20 check).

### Participant Recruitment

To assess eligibility, a pediatric psychiatrist will interview candidate participants using the Attachment Scale Interview (ASI) [73] to determine attachment style. Adolescents diagnosed as insecurely attached will be accepted in the study. Signed consent will be required from both parents and adolescents. The following inclusion and exclusion criteria will be used (Textbox 1).

### Information and Consent

Information packs will be given to adolescents on the day of the first meeting. Information packs will contain two detailed study statements: one for parents, and one for the adolescent. The adolescent’s consent form must be signed by both parents and by the adolescent. The participating parent must sign the parental consent form. The investigator will sign informed consent forms, with a copy for each participant, and the original stored by the investigator. Participants will be informed, in the participant’s information leaflet, that the data generated by the study will be accessible only to the investigator, or to the relevant health authorities for official inspection.

### Material

#### Oxytocin or Placebo Treatment

Syntocinon (NOVARTIS, Basel, Switzerland) is a synthetic, sterile, clear aqueous solution containing a cyclic nonapeptide, identical to endogenous OT released by the posterior lobe of the pituitary. The excipients are sodium acetate, glacial acetic acid, chlorbutanol, and ethanol. The PB will contain all the ingredients in the Syntocinon spray (saline solution) except for OT. Syntocinon and PB sprays, with identical ampoules and labels, will be prepared and randomly assigned by the research pharmacy at the University Regional Hospital Pharmacy of Besançon. Based on studies of IN-OT in adults [74], age-dependent dosage will be used, with age as a proxy for size and weight. Thus, younger participants (aged 13 to 15) will receive 16 international units (IU, 4 puffs of 4 IU), and older participants (aged 16 to 20) will receive 24 IU (6 puffs of 4 IU).

**Inclusion and exclusion criteria for participants.**

- Inclusion
  - Results of Attachment Scale Interview (ASI): clinical diagnosis of insecure attachment
  - Male adolescent
  - Enrolled in high school or college
  - Not hospitalized
  - No current or past history of neurological or psychiatric illness, including substance abuse or dependence
  - Aged 13 to 20 years
  - Able to speak and understand French
  - Accompanied by a parent
  - Normal or adequately corrected vision
  - Affiliation to French social security
  - Not participating in any other ongoing trials
  - Informed signed consent of adolescent and both parents
- Exclusion
  - Results of ASI: clinical diagnosis of secure attachment
  - Female
  - Intellectual deficit
  - Severe neurological symptoms
  - Known allergies to OT or to preservatives in the nasal spray (in particular, E216, E218 and chlorobutanol hemihydrates)
  - Outside of age range
  - Not speaking French (either adolescent or parent)
  - Vision problems
  - Chronic disease (ie, liver failure, kidney failure, or cardiovascular disease)
  - Antihypertensive therapy
  - Smoking
  - Heavy alcohol use or drug use
  - Participation in another ongoing trial

**Figure 1:**
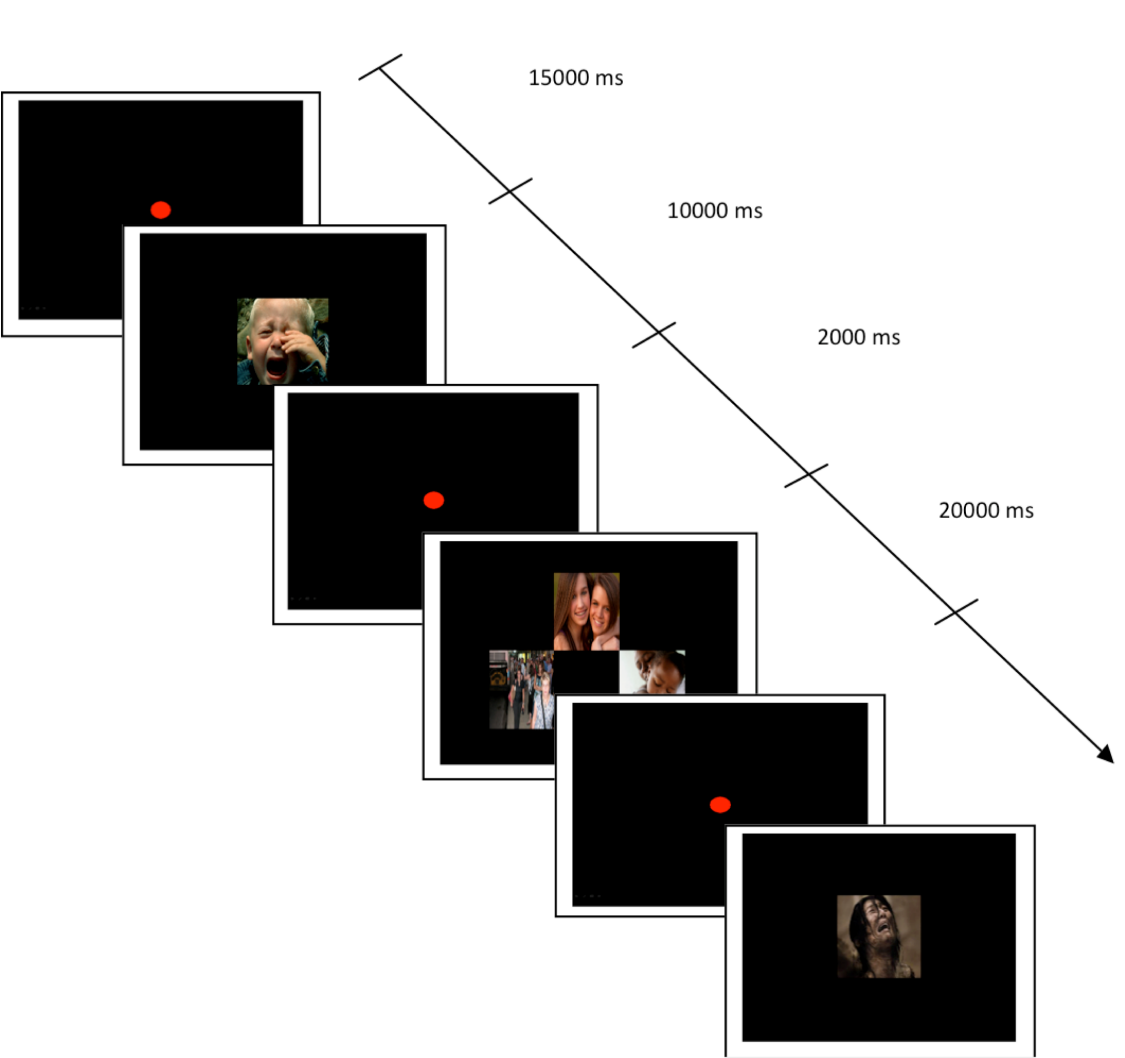
Examples of stimulus displays of the four picture categories (distress, comfort, joy-complicity, and neutral).

#### Stimuli

The Besançon Affective Picture Set-Adolescents (BAPS-Ado) [75] will be used to elicit attachment-related emotions. The following categories of emotional stimuli will be used: (1) “distress” (n=20), (2) “comfort” (n=20), (3) “joy-complicity” (n=20), and (4) “neutral” pictures (n=20). The first category of pictures is scenes of distress (ie, faces expressing sadness or anguish, or scenes of loss and separation). The second category is comfort-related scenarios (ie, a parent comforting an infant or an adolescent after an episode of distress). The third is pictures of complicity (ie, joyful moments such as parent-child interaction, and partner or peer interaction). The fourth category is neutral scenes (ie, persons walking along a street or in the subway). Levels of color saturation (50%) and lightness (50%) will be adjusted with Adobe Photoshop (Adobe Photoshop Elements 6.0, Los Angeles, USA). The pictures measuring 11.80 cm in height and 11.50 cm in width will be equiluminant and subtended 10.98° (horizontal) x 11.26° (vertical) of visual angle at a viewing distance of approximately 60 cm. All pictures will be displayed on a black background. Pictures will be pseudo-randomized in blocks. Each block will contain 1 slide with a picture of distress, displayed for 10 seconds, with an inter-stimulus-interval of 2 seconds, followed by 1 slide containing 3 pictures (comfort, joy-complicity, and neutral) presented simultaneously and displayed for 20 seconds. Each block of pictures will be separated by an inter-stimulus interval of 10 seconds (Figure 1).

### Eye-Tracking Apparatus

Eye-tracking (ET) data will be recorded using the Remote Eye-Tracking Device (RED), a non-invasive, contact-free, automatic ET and head movement compensation solution developed by SensoMotoric Instruments (SMI, Teltow, Germany). This system will capture data with temporal sampling at 250 Hz, spatial resolution of 0.03° and high accuracy of visual angle, 0.4°. The RED system will provide reliable binocular and pupil gaze data and allow subjects to wear glasses or contact lenses. The RED system, including Experiment Center 3.0 to control stimulus presentation, iView X 2.8 to control ET data acquisition, and BeGaze to record data, will be interfaced with a Dell laptop. Pictures will be presented on a stand-alone 20 inch monitor (1680 by 1050 pixel screen resolution) placed approximately 60 cm in front of the participant.

### Neurophysiological Measurement Apparatus

A BIOPAC 5 channel acquisition system (BIOPAC System Inc. Model MP 36, Goleta, CA) and a Dell Pentium computer will be used to collect neurophysiological data. AcqKnowledge 4.3 software (BIOPAC Systems, INC. Goleta, CA) will be used to obtain continuous recordings of the participant’s neurophysiological responses (EDA and HR). Two electrodes (BIOPAC Systems Inc., Model EL 507), placed on the second phalanges of the index and middle fingers of the non-dominant hand [76] will be used to record EDA. The EDA data will be digitized at 1000 samples per second, with a gain of 1000. Low (35 Hz) and high (.05 Hz) pass filters will be applied.

Electrocardiograms (ECG) will be collected, together with cardiac impedance, using a two-electrode (2560, 3 M RED DOTTM) configuration with the bio-impedance module for ground referencing. The electrodes will be connected to a BIOPAC ECG module with the gain set to 1000. The waveform will be used to estimate HR using the AcqKnowledge “Hemodynamics” function. HR will be calculated from the R-R intervals in an ECG.

### Attachment Style Assessment

Attachment will be assessed using a modified ASI, with questions adapted to be applicable to adolescents. This interview elicits the adolescent’s current state of mind regarding the quality of relational experiences with parents and peers. The ASI determines the adolescent’s ability to access and use social support with 3 confidants (ie, parents and peers). This tool assesses relationship quality, social support, and security of attachment style. Several dimensions (ie, mistrust, constraint of closeness, fear of rejection, self-reliance, desire for company, fear of separation, and anger) were coded to determine the attachment profile as either secure or insecure (ie, enmeshed, fearful, angry-dismissive or withdrawn).

### Parent-Adolescent Interaction Assessment

The quality of the parent-adolescent interaction will be assessed with the French version of the GPACS. The development of the GPACS drew on prior literature describing behavioral manifestations of security, insecurity, controlling behavior, and behavioral disorganization among younger children toward their parents in stressful situations. It has already been used in several studies [77-79]. The GPACS coding system includes the rating of each interaction on 10 5-point scales from 1 (not at all) to 5 (very much). There are 2 positive scales (1) “Collaborative Communication” indexing cooperative goal-corrected partnership and parent/adolescent carefulness; and (2) “Caregiving Validation of Adolescent’s Voice” indexing the caregiver’s support for the adolescent. There are 8 negative scales characterizing 3 subtypes of disorganized interaction (ie, punitive, caregiving/role confusion, and disoriented behavior). These 8 scales are (1) parental punitive behavior (eg, angry, critical or mocking comments about the adolescent); (2) adolescent punitive behavior (eg, angry, critical or mocking comments about the parent); (3) adolescent disoriented-distractible behavior (eg, suddenly stopping in midsentence and “freezing” with hand in midair or pausing abruptly in mi-sentence); (4) parental disoriented behavior (eg, the same as adolescent disoriented behavior); (5) adolescent odd, out-of-context or contradictory behaviors, which may seem disjointed, startling, or inexplicable to an observer (eg, using a forced, high-pitched, or childish tone of voice, shifting into unusual, fantasy-based topics); (6) parent odd, out-of-context behavior (eg, using a forced, high-pitched, or childish tone of voice, wandering around the room, stiff, usually shifting away from the topic); (7) adolescent attempts to manage or take care of the parent or modulate the parent’s behavior (eg, offering guidance, defusing tension with over-bright, entertaining behavior); and (8) parental role confusion (eg, asking for advice on topics typically discussed with partner or other adult).

### Psychological Evaluation

To evaluate psychiatric traits in participants we will use an abbreviated version of the Beck Depression Inventory (BDI) [80], the Spielberger State-Trait Anxiety Inventory (STAI, Forms Y A and Y B) [81], and the Toronto Alexithymia Scale (TAS-20) [82].

#### The Beck Depression Inventory

BDI-II will be used as a depression-screening tool. The BDI-II is a self-assessment instrument with 21 items that assess the presence and severity of depressive symptoms. Each question is scored 0 (symptom absent), 1 (symptom present), 2 moderate symptom), or 3 (severe symptom). The total potential score is 63.

#### The Spielberger State-Trait Anxiety Inventory Forms Y A and Y B

This self-report assessment indicates the intensity of feelings of anxiety; it distinguishes between state anxiety (a temporary condition experienced in specific situations) and trait anxiety (a general tendency to perceive situations as threatening). Responses for the S-Anxiety scale will assess intensity of current feelings “at this moment” from 1 (not at all), 2 (somewhat), 3 (moderately so), to 4 (very much so). Responses for the T-Anxiety scale will assess frequency of feelings “in general” from 1 (almost never), 2 (sometimes), 3 (often), to 4 (almost always). The scale has been standardized in French.

#### The Toronto Alexithymia Scale

The TAS-20 [82] comprises 3 factors to assess difficulties in identifying and describing feelings, which are thought to reflect a deficit in cognitive processing and regulation of emotional states [83].

### Eye-Tracking Measurements

The whole of each picture will be treated as a single area of interest (AOI). Prior to detailed statistical analyses, AOIs will be divided into 4 categories (distress, comfort, joy-complicity, and neutral) and each category will be analyzed separately. Fixations will be defined temporally and spatially, using a pre set minimum fixation duration of 80 milliseconds and a maximum dispersion value of 100 pixels. In order to explore different gaze patterns, various parameters (ie, fixation order, first fixation duration, fixation duration, number of fixations, and entry time) will be analyzed in relation to attachment style.

The mean fixation order of an AOI category will be calculated by averaging the fixation order of each AOI in the same category of pictures. The mean first fixation duration (ms) in the AOI will be calculated by averaging first fixation duration for each AOI in the same category of pictures. The mean duration of a fixation (ms) in the AOI will be calculated by averaging the fixation duration for each AOI in the same category. The mean number of fixations will be calculated by averaging the number of fixations in each AOI category. The mean sum of fixation duration will be calculated by averaging the sums of duration of all fixations in an AOI category. The mean entry time for an AOI will be determined by averaging entry time for each AOI in the same category.

### Neurophysiological Measurements

EDA will be measured by skin conductance level (SCL) and SCRs. The SCR will be defined as the maximum change in conductance (in µSiemens) in the 0.1 to 6 second window after stimulus onset. The change in neurophysiological response will be calculated by subtracting mean levels of neurophysiological response SCRs (ie, latency and amplitude) during the baseline from mean levels during each slide viewing, a practice commonly used in neurophysiological research [65,67]. HR will be calculated from the R-R intervals in the ECG.

### Procedure

**Figure 2:**
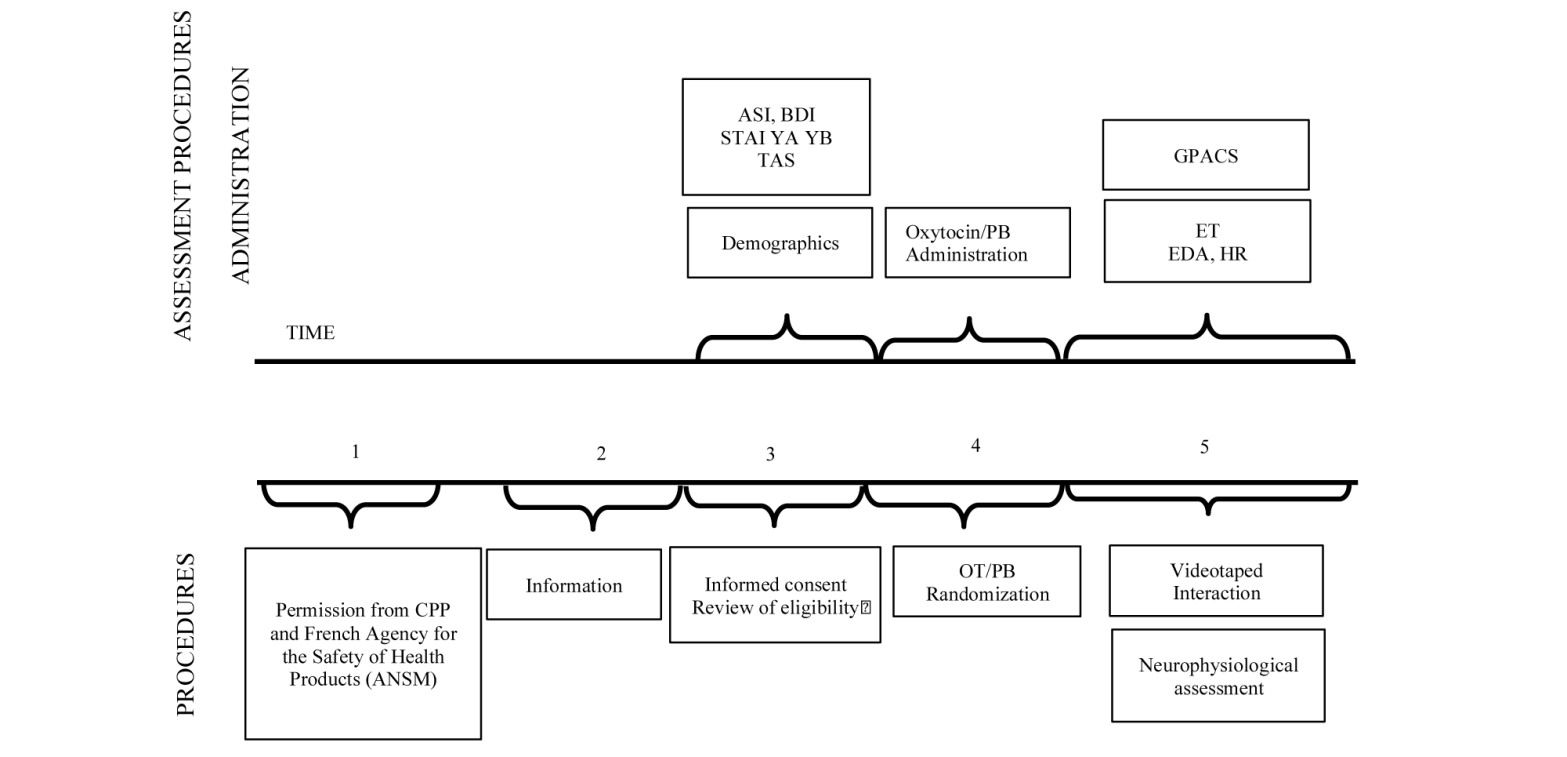
General study protocol.

First, a psychologist will evaluate the participant’s diagnostic eligibility. Attachment style will be determined using the ASI [74]. Participants who are classified as insecurely attached will return 1 week later for the OT trial (see Figure 2). Randomization will be centralized and performed after the inclusion of the eligible participants. Participants will be randomly assigned to receive either OT or PB intranasally, with both investigators and participants blind to condition. The randomization code will be kept secret by the pharmacy responsible for dispensing the corresponding medication. To reduce bias, randomization will be performed in blocks, with stratification for confounding factors (eg, age). Experiments will be conducted in a comfortable laboratory during the mid-afternoon hours, in accordance with previous research suggesting stability in the diurnal cycle of plasma OT [84]. Each adolescent will first be asked to choose a topic of disagreement to discuss during the parent-adolescent interaction. Then, 45 minutes before the interaction, the adolescent will be instructed to sit and self-administer 1 puff every 30 seconds, alternating nostrils. The adolescent will take each dose in front of the investigator, to assure correct administration and tolerability. The dosage and timing of the nasal spray administration were chosen based on published research on IN-OT, behavior, and emotions in humans [85], and results on cerebrospinal fluid levels after intranasal vasopressin administration [86]. Dyadic interaction will consist of a 5-minute free conversation and a 10-minute conflict discussion that will both be videotaped. The entire interaction session will be transcribed verbatim, and then coded offline for verbal interaction behavior with the GPACS by coders blind to the study hypotheses. After the dyadic stressful interaction, the adolescent will then be isolated in the ET room for 15 minutes, in order to adapt to experimental conditions. The adolescent will be seated in front of the screen in a comfortable viewing position. After this adaptation phase, the EDA electrodes will be fastened to the second phalanges of the index and middle fingers of the non-dominant hand and 2 ECG electrodes will be placed in a bipolar configuration on interior sides of the participant’s wrists [87]. The RED will perform a 9-point calibration procedure. Once this calibration has been successfully accomplished, the 3 minute baseline responses will be recorded with the BIOPAC system. The participant will be informed that the test is due to start, and that he will freely observe a series of 80 pictures. The neurophysiological monitoring AcqKnowledge 4.3 software will be synchronized with Experiment Center 3.0 (RED SMI) software by event markers representing the beginning of each picture. The participant’s eye movements and neurophysiological recordings (EDA and HR) will begin when the first red dot (approximately 0.95° of visual angle) appears. The task will last for about 15 minutes.

### Study Periods

Entry into the study (T0) is the first study period and will take place in agreement with the French Agency for the Safety of Health Products and the local ethics committee. The adolescents’ parents agree to be included and sign the informed consent to enter into the clinical active phase of the study. The ASI results will be assessed, taking into account that an insecurity score on the ASI is a prerequisite for entry into the study. The intermediate study period (1 to 24 months, T1) will involve the inclusion criteria described in Textbox 1. In the third (end) study period (30 months, T2) the AOI parameters and neurophysiological data will be analyzed for the adolescent population. The GPACS analyses will be carried out for dyads included in the study. The results will be valorized.

### Outcomes

#### Primary Outcome

The primary outcome is the evaluation of the changes in adolescent-parent interaction using GPACS in the OT versus PB conditions.

#### Secondary Outcome

The secondary outcome is the evaluation of the changes in oculomotor behavior and neurophysiological responses (ie, modification of EDA and HR [88]) in the OT versus PB conditions.

### Safety Procedure

#### Side Effects

Findings show that IN-OT produces non-detectable subjective changes in recipients, and is not associated with adverse outcomes when delivered in doses of 18 to 40 IUs for short-term use in controlled research settings [89].

#### Withdrawal of Individual Participants

Participants may withdraw from the study at any time for any reason and without any sanction. Researchers, after consulting with the principal investigator and the study coordinator, may also interrupt the treatment program if, in their opinion, continuing this treatment is prejudicial to the patient’s welfare. If a participant withdraws or is withdrawn from the study, follow-up at day 30 will be continued whenever possible.

#### Suspension of the Study

In cases where severe adverse events related to the administration of the treatment are suspected, the study will be interrupted and the researchers and coordinator will decide whether to continue.

#### Reporting of Adverse Events

Any adverse events reported spontaneously by the participant or observed by the researcher or the research team will be recorded on the case report form (CRF) designed for this purpose. The researcher will classify the intensity of said adverse events in accordance with a mild to severe scale, and the periodicity of the event will be classified by following a single occurrence to persistent scale (Textbox 2).

**The classification scheme for reporting the intensity and periodicity of adverse events.**

1. Intensity

  a. Mild: some discomfort, but not as such as to interrupt normal daily activity.

  b. Moderate: sufficient discomfort to reduce or notably affect normal daily activity.

  c. Severe: causing incapacity to work or perform normal daily activities.

2. Periodicity

  a. Single occurrence: just one event of limited duration.

  b. Intermittent: various episodes of an event, each of limited duration.

  c. Persistent, unlimited: an event that has persisted over time and is of indefinite duration.

For each adverse event, its relation to the medication taken, in the researcher’s opinion (definitive, probable, possible, improbable, none), as well as any action taken as a result, will be recorded on the data collection form. The occurrence of an adverse event that is fatal, potentially fatal or incapacitating, or that requires or prolongs hospitalization, or that provokes severe congenital anomalies will be recorded as a “severe” adverse event (SAE). All SAEs and unexpected adverse pharmacological reactions, defined as adverse events whose nature or intensity is not in accordance with any expected adverse event, will be reported by the researcher to the study coordinator by telephone, mail or fax as soon as is reasonably possible, but in any case within 24 hours of occurrence.

### Data Analysis

#### Statistical Power, Establishment of Sample Size, and Safety

The calculation of sample size will be based on the average score on the 4 negative dimensions (items) of the GPACS between the group of adolescents who will receive treatment with OT and the group that will receive the PB. No clinical studies have used this scale in an attachment study, so this calculation is based on expert opinion (personal communication from K Lyons-Ruth, author of the GPACS). We estimate that a difference of 5 points for our main criterion is an interesting minimum clinical difference. Based on the supposition of a difference of 5 points for the OT group, a standard deviation value of 6.2 was calculated. We do not have the standard deviation of the values on our primary endpoint. However, we have the standard deviation of each negative item in the GPACS and summarized data from the original validation study of the GPACS. The variability of the values obtained for each of the 4 negative items were summed and we obtained a variability of 0.03 (SD 0.175). If we consider that the sum represents 3% of the total variability (co-variations to be added), then we arrive at a standard deviation of 6.2, a 90% power and an alpha level of 5% unilateral (superiority of trial against PB); therefore 54 patients should be included. Taking into account 10% of error and non-analyzable data, 60 patients should be included (ie, 30 patients per group). The overall significance level of statistical tests will be at 5%.

#### Statistics

To analyze the quantitative variables, parametric (Student *t*) or nonparametric (Wilcoxon) tests will be used, depending on the distribution of variables. The Shapiro-Wilk test will be used to test the normality of the distributions. Clinical and sociodemographic variables collected at the beginning of the study will be described using the mean (SD) for normally distributed continuous variables, and the median, for non-normally distributed variables. For qualitative variables, the chi-square or Fisher's test will be used to compare proportions between the two groups. Statistical analyses will be conducted and supervised by the Methodology and Biostatistics cell of the Centre of Investigation Clinic (CIC) in Besançon. All data will be analyzed using the SAS application. To estimate the clinical relevance of the findings, Cohen’s effect size (Cohen’s d) will be used for parametric outcomes (large effect ≥ 0.8) and *r* for non-parametric outcomes (large effect ≥.5).

## Results

Enrollment for the study will be completed in May 2016. Data analysis will commence in July 2016. Study results are to be published in the winter of 2017.

## Discussion

### Principal Findings

In order to investigate the potential clinical implications of OT as an add-on treatment in psychotherapy, it is necessary to establish a well-designed, randomized, controlled protocol to assess the impact of OT on emotion regulation skills in adolescents. Research shows that OT might induce a momentary emotional state of trust and safety, while enhancing self-confidence. Administration of a single dose of OT might ameliorate emotional regulation skills in adolescents, and thus promote their capacity to connect with parents and peers. Given that adolescence is a critical period for the refinement of interpersonal and intrapersonal competences in close relationships, emotion regulation skills in conflict situations may have direct implications for experiences in close friendships and romantic relationships. Experimental research on OT and emotion regulation in adulthood has provided a scientific basis for the administration of OT, and has already had a positive impact on many short- and long-term health outcomes. The proposed research should contribute significantly to the understanding of the role of OT in emotion regulation, and represents a critical domain that might contribute significantly to improving health and well-being. This protocol could also be used in clinical interventions to increase positive life outcomes for insecure adolescents. Additionally, IN-OT may hold promise for the therapeutic neuroenhancement of parent-adolescent close relationships.

A multi-dimensional approach should prove valuable in characterizing the intra- and inter-individual differences underlying both emotions and attachment, and their impact on social function. Studying attachment-related emotion regulation via oculomotor assessment and neurophysiological strategies during emotional picture viewing might provide additional evidence for the implicit defensive and/or approach strategies of emotion regulation.

Methods traditionally employed in emotion regulation studies [90] require conscious effort and self-monitoring processes from participants. In this study, participants will not be instructed to exert effortful regulation of their emotions. We seek to investigate emotion regulation mechanisms that will be automatically triggered by the stimulus itself and will take place without monitoring or awareness [91].

### Limitations

This is the first correlational study of a healthy adolescent population that explicitly investigates the role of OT in stressful interaction, combined with analysis of oculomotor behavior and neurophysiological reactions in attachment-related pictures (the BAPS [75]). There are, however, some potential limitations (1) only one dose of OT is administered; (2) only the healthy male adolescent population is studied; and (3) only static images are used.

Future investigation should seek to determine the effects of higher doses, administered in both clinical and healthy populations. Researchers should also examine the effects of OT on emotion regulation with different types of social support (eg, peers or strangers). Dynamic stimuli could also be used for ET studies, as they provide more naturalistic interpersonal situations than static pictures. Gaze pattern modulation could then be analyzed over time. Given the central role of OT in human social relationships, further research should integrate longitudinal data on the biological and family and/or environmental systems, focusing on triadic interactions between the healthy male adolescent and both of his parents.

## Abbreviations

AOI: area of interest
ASI: Attachment Scale Interview
BAPS-Ado: Besançon Affective Picture Set-Adolescents
BDI-II: Beck Depression Inventory-II
EDA: electrodermal activity
ECG: electrocardiogram
ET: eye tracking
GPACS: Goal-Corrected Partnership in Adolescence Coding System
HR: heart rate
IN-OUT: intranasal oxytocin
IU: international unit
OT: oxytocin
PB: placebo
RED: remote eye-tracking device
SAE: severe adverse event
SCL: skin conductance level
SCR: specific conductance response
TAS-20: Toronto Alexithymia Scale-20
